# Kurt Goldstein (1878–1965)

**DOI:** 10.1007/s00415-013-7020-1

**Published:** 2013-07-18

**Authors:** Stephen Pow, Frank W. Stahnisch

**Affiliations:** Department of Community Health Sciences, Hotchkiss Brain Institute, Institute for Public Health, University of Calgary, 3280 Hospital Drive N.W., Calgary, AB T2N4Z6 Canada


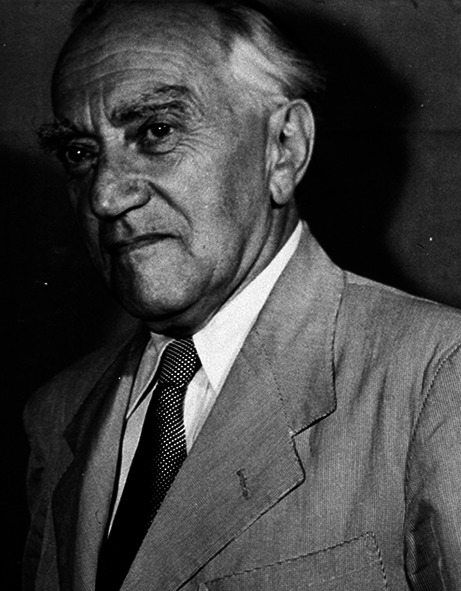
 In a recent commentary about “neural reuse,” a commentator noted that the implications of these theories for the treatment of brain injuries have been foreshadowed in the writings of the German-American neurologist Kurt Goldstein (1878–1965) [1]. Goldstein conceived his holistic approach to the brain, in which he postulated that function in a damaged area could be compensated through the capacity of other areas, in the 1930s without the benefits of modern technology. It is a testimony to his intuition that Goldstein’s ideas on neurology, psychology and rehabilitation not only remain relevant but receive vindication in the 21st century.

Goldstein was born to a Jewish family in Katowice (Silesia), then part of Germany (now Poland). Scholarly from a young age, he showed inclinations toward the natural sciences as well as the humanities. His father, a lumber merchant, tried to dissuade Goldstein from pursuing the “breadless art” of philosophy, and after only a year at Heidelberg University, Goldstein decided to pursue medicine, something he later attributed to humanitarian feeling:

Medicine appeared to me best suited to satisfy my deep inclination to deal with human beings and to be able to help them. The vague knowledge I had of medicine concerned mainly diseases of the nervous system, which seemed to me to be particularly in need of attention [3].

The Central-European neuroscience milieu of that time paid little attention to rehabilitation of brain-injured patients. Studying at the University of Breslau, where he obtained his M.D. in 1903, Goldstein became acquainted with the prevailing reductionist thinking in neurology, well-represented by his supervisor, aphasia researcher Carl Wernicke (1848–1905). Efforts to map the localized functions of the nervous system reflected the scientific trends of the 19th century. In addition, the ideas of the influential Munich psychiatrist Emil Kraepelin (1856–1926) on the critical role of inheritance in mental illness led many neuroscientists to believe that diagnosis and nosology were their chief aims [6].

During his residency at the Psychiatric Hospital of the University of Koenigsberg between 1906 and 1914, Goldstein began to challenge the so-called neurological ‘diagram makers’ and their theories of localized function. It would be incorrect to think that Goldstein arrived at his emerging holism without intellectual antecedents. He was especially influenced by the British neurologist John Hughlings Jackson (1835–1911) and his concepts of negative symptoms related to local damage and positive symptoms caused by modifications of surrounding areas [2]. Also apparent in Goldstein’s work is the ‘diaschisis’ notion of the Swiss-German neuroanatomist Constantin von Monakow (1853–1930), according to which damage to a given part of the nervous system can cause dysfunction in distant regions, through a type of stunning phenomenon [8].

In 1914, Goldstein had just moved to the laboratory of the neuroanatomist Ludwig Edinger (1855–1918) at the University of Frankfurt when the outbreak of World War I led to the establishment of specialized military hospitals. From 1916, Goldstein was head of the Frankfurt Institute for Research into the Consequences of Brain Injuries (*Institut zur Erforschung der Folgeerscheinungen von Hirnverletzungen*). In association with his friend, the experimental psychologist Adhémar Gelb (1887–1936), he devised rehabilitation programs aimed at returning brain-injured soldiers to some level of productivity. This was the formative period of Goldstein’s career, in which he designed a research program using innumerable case studies of war-injured soldiers to offset the therapeutic nihilism of the period. The success of the rehabilitation project resulted to a large extent from the interplay between Gelb’s talent to arrange the experiments and Goldstein’s ability to ask broad questions. Goldstein’s entire team was characterized by a wide-ranging interdisciplinarity that amalgamated Gestalt psychology, holist philosophy, and the latest neurophysiological techniques [9].

In 1930, Goldstein accepted the chairmanship of the Neurology Clinic at the academic hospital Berlin Moabit. However, his vision of a clinic that would practice as well as promote his notions of holism was dashed a few years later when the Nazis took power. Moabit had a reputation for being Jewish (as were 70 % of its physicians) as well as socialist, so it was an obvious target for the new government. A truckload of SA men showed up on April 1st, 1933 to take Goldstein into custody. When he delayed following them because there was no physician to take over his rounds, an SA man shouted, “Everyone can be replaced, including you!” [5].

Goldstein was imprisoned and tortured while his assistant (and later spouse) Eva Rothmann (1897–1960) petitioned the high-ranking Nazi, Matthias Heinrich Goering (1879–1945), who was a psychologist by training, for his release. This was granted on the condition that Goldstein would leave Germany forever—a demand with which he promptly complied. Taking up an ad hoc position at the University of Amsterdam from 1933 to 1934, made possible by a Rockefeller fellowship, he wrote his masterpiece, *Der Aufbau des Organismus* (‘*The Organism*’):

It has been found that, even in cases of circumscribed cortical damage, the disturbances are scarcely ever confined to a single field of performance […]. The relationship between mental performances and definite areas in the brain constitute a far more complicated problem than the so-called localization theory has assumed [4].

Subsequently, Goldstein immigrated to the USA, where he fulfilled a number of academic roles. He was professor of neurology at Columbia from 1936 to 1940, and in 1938 he delivered the William James lectures at Harvard. From 1940 to 1945 he had a position at Tufts in Boston together with a neuropsychiatry practice, while he continued to lecture at City University of New York from 1950 to 1955. In his late seventies he still taught courses at Brandeis University. Yet his last years were marred by tragedy: his wife committed suicide after a long illness and for Goldstein himself, America remained a foreign country. He died in 1965 after a fall that resulted in aphasia, a condition he had always closely researched [7].

## References

[1] Anderson M (2010). Neural Reuse: a fundamental organizational principle of the brain. Behav Brain Sci.

[2] Goldstein G (1990). Contributions of Kurt Goldstein to Neuropsychology. Clin Neuropsychol.

[3] Goldstein K, Riese W (1967). Autobiography. A history of psychology in autobiography.

[4] Goldstein K (1939). The organism: a holistic approach to biology derived from pathological data in man.

[5] Harrington A (1998) Kurt Goldstein’s neurology of healing and wholeness: A Weimar story. In: Greater than the parts: holism in biomedicine. Oxford, Oxford University Press, p 39

[6] Harrington A (1996). Reenchanted science.

[7] Noppeny U (2000). Abstrakte Haltung: Kurt Goldstein im Spannungsfeld von Neurologie, Psychologie und Philosophie.

[8] Noppeny U, Wallesch C (2000). Language and cognition—Kurt Goldstein’s theory of semantics. Brain Cogn.

[9] Stahnisch F, Hoffmann T (2010) Kurt Goldstein and the neurology of movement during the interwar years. In: Hoffmann C (ed) Was bewegt uns? Projekt Verlag, Bochum, pp 283–312

